# Perception of facial and dental asymmetries and their impact on oral health-related quality of life in children and adolescents

**DOI:** 10.1007/s00056-023-00490-2

**Published:** 2023-08-28

**Authors:** Katharina Flanze, Sandra Riemekasten, Christian Hirsch, Till Koehne

**Affiliations:** 1 Department of Pediatric Dentistry, University Medical Center Leipzig, Leipzig, Germany; 2 Department of Orthodontics, University Medical Center Leipzig, Liebigstraße 12/1, 04103 Leipzig, Germany

**Keywords:** Facial asymmetry, Midline deviation, Stereophotogrammetry, Soft tissue assessment, Malocclusion, Gesichtsasymmetrie, Mittellinienabweichung, Stereophotogrammetrie, Assessment von Weichgewebe, Malokklusion

## Abstract

**Background:**

The aim of this study was to investigate the perception of facial and dental asymmetries in children and adolescents and how these asymmetries affect their psychosocial and emotional well-being.

**Methods:**

The study included 66 children and adolescents (7–15 years) with a deviation between the maxillary and mandibular dental midlines of > 0.5 mm. The soft tissues of the face were scanned using stereophotogrammetry. Psychosocial and emotional impairments were assessed using the German version of the Child Perceptions Questionnaire (CPQ-G8-10 and 11–14).

**Results:**

The mean midline deviation of the study group was 2.3 mm with no significant gender differences. Girls perceived facial asymmetry significantly more often than boys (*p* < 0.01). However, stereophotogrammetry showed no significant differences in facial morphology between subjects who perceived their face as asymmetrical and those who perceived it as symmetrical. Interestingly, we observed a significant correlation between the deviation of the dental midline and the lateral displacement of gonion (*p* < 0.05) and cheilion (*p* < 0.01). Psychosocial and emotional impairment was significantly higher in girls than in boys (*p* < 0.05). However, there was no significant correlation with the measured facial asymmetries. In contrast, the CPQ subscale score was 2.68 points higher in individuals with a dental midline shift ≥ 3 mm (*p* < 0.01), independent of age and gender.

**Conclusion:**

Although girls perceived facial asymmetries more strongly than boys do, this perception could not be objectified by extraoral measurements. A midline shift of 3 mm or more had a negative impact on the oral health-related quality of life of affected children and adolescents.

**Supplementary Information:**

The online version of this article (10.1007/s00056-023-00490-2) contains supplementary material, which is available to authorized users.

## Abstract (de)

**Hintergrund:**

Ziel dieser Studie war es, die Wahrnehmung von fazialen und dentalen Asymmetrien bei Kindern und Jugendlichen zu untersuchen und zu ermitteln, wie diese Asymmetrien ihr psychosoziales und emotionales Wohlbefinden beeinflussen.

**Methoden:**

Die Studie umfasste 66 Kinder und Jugendliche (7-15 Jahre) mit einer Abweichung von > 0,5 mm zwischen der oberen und unteren Zahnmittellinie. Die Weichteile des Gesichts wurden mittels Stereophotogrammetrie gescannt. Psychosoziale und emotionale Beeinträchtigungen wurden mit der deutschen Version des Child Perceptions Questionnaire (CPQ-G8-10 und 11-14) erfasst.

**Ergebnisse:**

Die mittlere Mittellinienabweichung der Studiengruppe betrug 2,3 mm, ohne signifikante geschlechtsspezifische Unterschiede. Mädchen nahmen signifikant häufiger eine Gesichtsasymmetrie wahr als Jungen (*p* < 0,01). Die Stereophotogrammetrie zeigte jedoch keine signifikanten Unterschiede in der Gesichtsmorphologie zwischen Probanden, die ihr Gesicht als asymmetrisch wahrnahmen, und solchen, die es als symmetrisch empfanden. Interessanterweise beobachteten wir eine signifikante Korrelation zwischen der Abweichung der Zahnmittellinie und der seitlichen Verschiebung von Gonion (*p* < 0,05) und Cheilion (*p* < 0,01). Die psychosoziale und emotionale Beeinträchtigung war bei Mädchen signifikant höher als bei Jungen (*p* < 0,05). Es bestand jedoch keine signifikante Korrelation mit den gemessenen Gesichtsasymmetrien. Im Gegensatz dazu war der CPQ-Subskalenwert bei Probanden mit einer Verschiebung der Zahnmittellinie ≥ 3 mm um 2,68 Punkte höher (*p* < 0,01), unabhängig von Alter und Geschlecht.

**Schlussfolgerung:**

Zwar nahmen Mädchen Gesichtsasymmetrien stärker wahr als Jungen, doch konnte diese Wahrnehmung nicht durch extraorale Messungen objektiviert werden. Eine Mittellinienverschiebung von 3 mm oder mehr hatte einen negativen Einfluss auf die mundgesundheitsbezogene Lebensqualität der betroffenen Kinder und Jugendlichen.

## Introduction

Aesthetic perception (Greek: aisthánesthaí = to perceive) is the main reason why patients consider orthodontic treatment [4]. However, the function of the dentition is usually the guide for determining the medical necessity of orthodontic treatment [32]. The patient’s perception and social needs play a subordinate role here [1].

Sensory impressions and the interpretation of these impressions determine our perception of the body. Importantly, this perception changes with age, as interpreting sensory input depends on cognitive development [27]. In fact, adolescents become increasingly critical of their physical characteristics and the importance of social acceptance increases [10]. This is also often the age at which perceptions of physical features no longer necessarily correspond to anatomical reality [33].

Health is defined as a state of complete physical, mental, and social well-being [3, 39]. This definition is valid across age groups and indeed even 8‑year-olds understand health as a conglomerate of not only somatic, but also emotional and psychosocial aspects [26, 29]. Importantly, oral health-related quality of life is an integral part of health-related quality of life [32]. Accordingly, dental abnormalities perceived by children and adolescents as requiring treatment can lead to psychosocial impairment and reduce oral health-related quality of life [9, 11]. Numerous studies have demonstrated the importance of various dental malocclusions for oral health-related quality of life [15, 18]. However, the relationship between dental midline shifts and facial asymmetries has not been investigated. Symmetry plays an important role in the perception of aesthetics, as humans are sensitive to disturbances in a bilaterally symmetrical pattern such as the face [38]. As the oral area is the focus of attention in communication, anterior symmetry is also of essential importance and the patient’s perception is particularly sensitive here [25].

The aim of this study was to investigate the extent to which patient perceptions of facial asymmetries are consistent with clinical measurements, such as three-dimensional (3D) facial photography, and the extent to which facial and dental asymmetries influence the psychosocial and emotional well-being of children and adolescents.

## Materials and methods

### Study stetting and participants

**Fig. 1:**
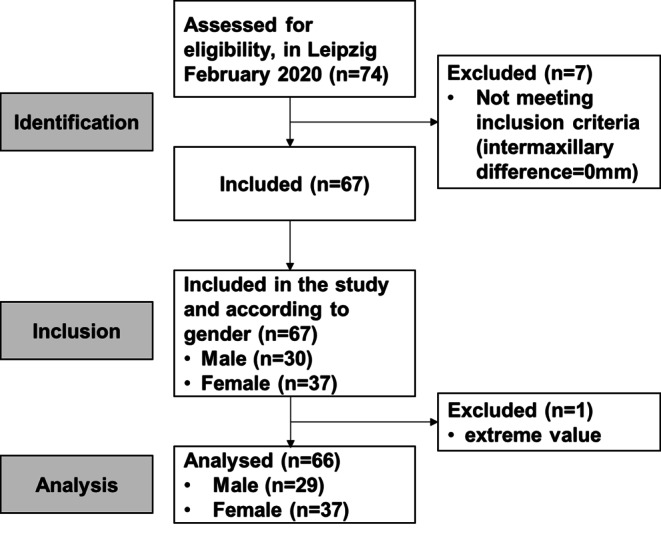
Strengthening the Reporting of Observational Studies in Epidemiology (STROBE) flowchart of eligible participants STROBE(Strengthening the Reporting of Observational Studies in Epidemiology)-Flussdiagramm zu geeigneten Probanden

The study took place between February and July 2020 and included 74 children and adolescents (41 girls and 33 boys) who had a midline shift of more than 0.5 mm between the upper and lower dental arches. The participants were patients of the Department of Orthodontics at the University of Leipzig who presented for orthodontic treatment for the first time. Figure 1 shows the flowchart according to the STROBE criteria. Finally, 66 children and adolescents (37 girls and 29 boys) aged 7–15 years were selected. The mean age was 11.4 years, with no significant difference between the sexes (Table 1).

**Table 1:** Description of study population regarding gender Beschreibung der Studienpopulation in Hinblick auf das Geschlecht

		Total (*N* = 66)	Female (*N* = 37)	Male (*N* = 29)	*p*
Mean age, years (± SD)	–	11.4 (1.9)	11.5 (1.8)	11.2 (2.0)	n. s.
Mean midline difference, mm (± SD)	–	2.27 (1.23)	2.34 (1.23)	2.17 (1.24)	n. s.
Age group, % (*N*)	≤ 11	48.5 (32)	48.6 (18)	48.3 (14)	n. s.
	> 11	51.5 (34)	51.4 (19)	51.7 (15)	
Mean dmft (± SD)	–	1.7 (2.7)	1.6 (2.4)	1.9 (3.1)	n. s.
Perceived difference in facial halves, % (*N*)	Yes	39.4 (26)	54.0 (20)	20.7 (6)	0.004
Mean score psychosocial and emotional impairment (± SD)	–	3.14 (4.10)	4.14 (4.13)	1.86 (3.74)	< 0.05

*SD* standard deviation, *dmft* decayed, missing, filled teeth, *n.s.* not significant

**Fig. 2:**
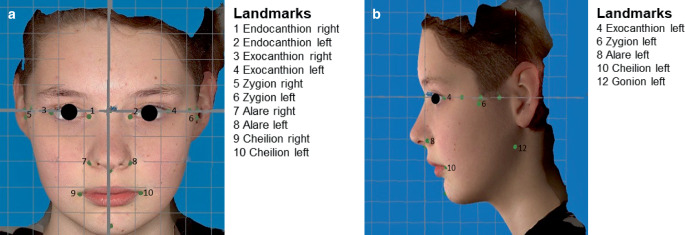
Three-dimensional (3D) analysis of the facial surface with the studied landmarks in the Cartesian coordinate system (Vectra Analysis Module): (**a**) frontal view; (**b**) lateral view Dreidimensionale (3-D)-Analyse der Gesichtsoberfläche mit den untersuchten Landmarken im kartesischen Koordinatensystem (Vectra Analysis Module): (**a**) Frontalansicht; (**b**) Seitenansicht

The study was approved by the ethics committee of the University of Leipzig (346/18-ek). The aim and procedure of the study were explained to the children and parents, and written informed consent was obtained. In addition, one person gave written consent for the publication of her facial photographs (Fig. 2).

### Questionnaire

To assess patients’ perceptions of dental and facial asymmetry, subjects completed a modified version of the Child Perceptions Questionnaire (CPQ-G8-10 or 11-14). The german Child Perceptions Questionnaire is a culturally adapted counterpart to the North American CPQ-8-10 and 11-14 [2]. The questionnaire is age-specific [3] and divided into four dimensions (oral symptoms, functional limitations, emotional and social well-being) [3]. Children are asked to rate their oral health-related quality of life over the past 3 months using a five-point scale representing the severity of impairment [2]. The response options ’never’, ’hardly ever’, ’sometimes’, ’often’ and ’very often’ correspond to a score between 0 and 4 and are combined to form a total or scale score. Low scores indicate high well-being (few limitations) and vice versa [3]. For the present analysis, we used the questions of the subscale ’emotional and social well-being’ to describe QoL impairments. For specific study purposes, the following dichotomous question was added to the questionnaire: “Have you ever noticed that the right and left sides of your face are different?”

## Clinical examination

### Dental midline shift and caries index

The dental midline displacements between the maxillary and mandibular arches were measured with a millimeter gauge during the initial orthodontic examination. The contact points of the upper and lower central incisors were used as reference points for the measurement. Analysis was performed using the sum of the measured values. Dental caries was scored according to World Health Organization (WHO) criteria and reported as a summary score (dmf-t + DMF-T: decayed, missing, filled teeth) in mixed dentitions.

### Three-dimensional photography

To record facial morphology, an image of each subject was taken using the VECTRA®H2 system (Canfield Scientific, Parsippany, NJ, USA; Canfield Scientific GmbH, Bielefeld, Germany). The VECTRA®H2 system is a portable stereophotogrammetric camera system based on passive stereophotogrammetry. This method provides a geometrically precise recording and display of the facial surface (soft tissue profile) in three-dimensional space [14]. The subjects were asked to assume their final bite position during the recording. The Vectra®H2 system uses a Canon EOS 800D (EOS Rebel T7i). The effective number of megapixels is 24.2 MP. The maximum image resolution is 6000 × 4000 MP [7]. To create a virtual 3D model, three separate photographs were taken (two lateral from diagonally below and one frontal). The acquisition time of a photo is 2 ms and the geometric resolution is 0.9 mm (triangulation) [34]. Data analysis of the 3D models is performed by the integrated Vectra Analysis Module (VAM) software [34]. Defined reference points were set in the face scans [12]. These landmarks can be reliably located and are homologous [5]. In line with international studies, we focused on the following six prominent bilateral landmarks (Fig. 2a, b, [31]):

- Exocanthion (Ex) right and left,
- Endocanthion (En) right and left,
- Zygion (Zyg) right and left,
- Alare (Ala) right and left,
- Cheilion (Chei) right and left, and
- Gonion (Go) right and left.

The symmetry analysis of the 3D face surface was based on linear distances and vector calculation [30]. The midline defined by the nasion and subnasal landmarks took the function of the median-sagittal plane of symmetry (y-axis) [12]. The distances of the landmarks from the mirror plane (x-values) were determined in both hemispheres. Two measurements were taken at the x‑coordinates: The difference between the distances of the two corresponding landmarks to the symmetry plane, which indicates the absolute symmetry in mm, and the symmetry index, which indicates the percentage ratio of the sum of the two distances (right and left). The symmetry index is therefore a dimensionless characteristic that is 50% for absolute symmetry. For an offset in three-dimensional space (x, y, z coordinates), an additional vector was calculated for the symmetry comparison of a bilateral pair. Therefore, the three-dimensional coordinates of each landmark were subtracted. The magnitude of the newly created vector was used as a reference for the asymmetry in three-dimensional space.

### Statistical evaluation

We assessed individuals for severity of facial asymmetry and deviation between the upper and lower dental midline, as well as for gender, age, and psychosocial and emotional impairment scores. We compared perceived and actual metric facial asymmetries, recorded using the Vectra H2® (Canfield Scientific, Inc., Parsippany, NJ, USA) system. We also performed a linear regression analysis with the deviation between the upper and lower dental midline as the independent variable and the respective landmarks (two- and three-dimensional parameters) as the dependent variable.

The two-sample t‑test was used to test whether the asymmetry values of the respective landmarks differed between the different perception groups (yes/no response). We also differentiated between gender and age using analysis of variance (ANOVA). For comparison of the age groups we chose the limit of 11 years in accordance with the CPQ-G8-10 and CPQ-G11-14.

The CPQ‑G score for the psychosocial and emotional well-being subscales was plotted with a 95% confidence interval (95% CI) for the criteria of age and midline difference, taking into account gender. Multivariable linear regression was used to analyze the effect of midline shift on psychosocial and emotional impairment, controlling for gender and age. Coefficients, 95% confidence intervals and *p*-values were calculated for all independent variables.

## Results

### Dental midline deviations correlate with displacement of gonion and cheilion

The range of displacement between the upper and lower midline was between a minimum of 0.5 mm and a maximum of 6 mm, with an average of 2.27 mm (standard deviation [SD] ± 1.23) mm. No gender differences were observed (Table 1). The mean dmf‑t value was 1.7 (SD ± 2.7) and showed no association with gender or midline deviation (Tables 1 and 3). The absolute metric values (x values in the coordinate system) of the cheilion (mouth angle) and gonion (jaw angle) correlated significantly with the midline deviations (Table 2). Patients with a midline deviation of ≥ 3 mm also had a significantly greater lateral displacement of gonion on the x‑axis (Go, *p* = 0.04). There were also significant correlations between some soft tissue landmarks (Table 2).

**Table 2:** Correlation coefficients of dental and facial asymmetry (x-axis) Dentale und faziale Asymmetrie (x-Achse), Korrelationskoeffizienten

	Intermaxillary difference (mm)	Ex	En	Zyg	Ala	Chei
Ex	0.09	–	–	–	–	–
En	0.01	0.60**	–	–	–	–
Zyg	0.02	−0.06	−0.05	–	–	–
Ala	−0.06	0.18	0.13	0.01	–	–
Chei	−0.33**	0.04	0.08	−0.11	0.05	–
Go	0.27*	−0.10	−0.27*	0.15	−0.30*	−0.32**

*Ex* Exocanthion, *En* Endocanthion, *Zyg* Zygion, *Ala* Alare, *Chei* Cheilion, *Go* Gonion

* *p* < 0.05

** *p* < 0.01

**Table 3:** Comparison of individuals with less or more/equal than 3 mm midline deviation Vergleich von Individuen mit weniger bzw. mehr als/gleich 3 mm Mittellinienabweichung

Midline deviation		< 3 (*N* = 43)	≥ 3 (*N* = 23)	*p*
Mean age, years (± SD)	–	11.5 (2.0)	11.1 (1.9)	n. s.
Gender, % (*N*)	Male	46.5 (20)	39.1 (9)	n. s.
	Female	53.5 (23)	60.9 (14)	
Age group, % (*N*)	≤ 11	46.5 (20)	52.2 (12)	n. s.
	> 11	53.5 (23)	47.8 (11)	n. s.
Mean dmft (± SD)	–	1.3 (2.0)	2.5 (3.6)	n. s.
Perceived difference in facial halves, % (*N*)	Yes	37.2 (16)	43.5 (10)	n. s.
Mean psychosocial and emotional impairment (± SD)	–	2.16 (2.94)	4.96 (5.26)	< 0.01

*SD* standard deviation, *dmft* decayed, missing, filled teeth, *n.s.* not significant

### Perceived asymmetry not correlated with facial measurements

In all, 39.4% (*n* = 26) of the subjects perceived differences in their half of the face in a right-left comparison (Table 1). Girls perceived asymmetry of the face significantly more often than boys (*p* = 0.004).

**Fig. 3:**
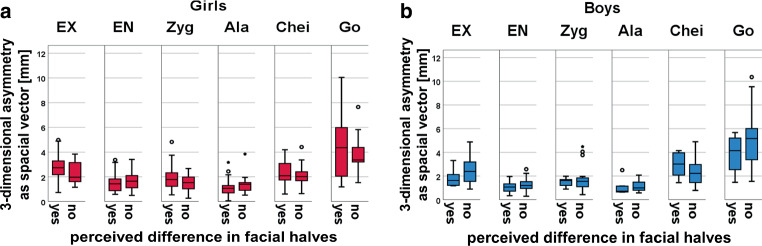
Three-dimensional (3D) analysis of the surface of the face with the landmarks studied: (**a**) girls’ perception of differences in face halves; (**b**) boys’ perception of differences in face halves. Abbreviations defined in Table 2 Dreidimensionale (3-D) Analyse der Gesichtsoberfläche mit den untersuchten Landmarken: (**a**) Wahrnehmung der Unterschiede zwischen den Gesichtshälften durch Mädchen, (**b**) Wahrnehmung der Unterschiede zwischen den Gesichtshälften durch Jungen. Abkürzungen s. Tab. 2

To correlate self-perceived facial asymmetry with facial anatomy, we next compared the 3D face scans of the perception groups (those who perceived facial asymmetry themselves versus those who did not). No significant differences were found in objective asymmetry (for all landmarks *p* > 0.05), either in the two-dimensional frontal plane (symmetry index and absolute asymmetry) or in three-dimensional space (position vector in three-dimensional space; Fig. 3). Furthermore, there were no significant differences in facial asymmetry scores (frontal plane and three-dimensional) between girls and boys (for all landmarks *p* > 0.5; Supplementary Fig. 1).

### Dental midline deviation magnitude vs. psychosocial and emotional well-being

The mean score for the psychosocial and emotional dimensions of the CPQ‑G was 3.14 points (SD ± 4.10), with a maximum score of 15 points (Table 1). The mean score for girls was more than twice as high as that for boys (Table 1). This difference was statistically significant (*p* < 0.05).

There was no significant relationship between facial asymmetry scores and psychosocial and emotional impairment. However, we observed a significant relationship between dental midline deviation and psychosocial and emotional impairment. In fact, subjects with dental midline deviations ≥ 3 mm had significantly higher psychosocial and emotional impairment than the comparison group with midline deviations < 3 mm (*p* = 0.007; Table 3).

Multiple linear regression analysis confirmed a significant influence of gender and midline shift on the psychosocial and emotional dimensions of the CPQ (Table 4). Each additional millimeter of midline shift was associated with an increase in impairment of approximately 1 scale point. There was no additional effect of the subject’s age (Table 4).

**Table 4:** Multiple linear regression analysis of the effect of intermaxillary difference, age, and gender on psychosocial and emotional impairment Multiple lineare Regressionsanalyse der Auswirkung von Intermaxillardifferenz, Alter und Geschlecht auf psychosoziale und emotionale Beeinträchtigungen

	Psychosocial and emotional impairment		
	Coefficient	95% confidence interval	*p*
Gender (female)	2.06	0.16 to 3.96	< 0.05
Age group (≤ 11/> 11 years)	0.65	−1.22 to 2.53	n. s.
Midline difference (< 3/≥ 3 mm)	2.68	0.71 to 4.65	< 0.01

## Discussion

Our results show that the presence of dental asymmetry significantly worsened the oral health-related quality of life of affected children and adolescents, regardless of age and gender, especially when the dental midline shift was greater than 3 mm. In contrast, we found no significant correlation between measured extraoral soft-tissue asymmetries and oral health-related quality of life. We explain this contradictory finding by the fact that there is no strong unidirectional correlation between facial asymmetry and midline deviation. Another important reason for our finding may be that the perception of extraoral asymmetries is highly subjective, especially in girls, regardless of the objective clinical findings.

Several studies have shown that malocclusion affects oral health-related quality of life [11]. However, none of these studies investigated the impact of dental midline discrepancies. Our analysis revealed an association between dental midline shift and psychosocial and emotional impairment in oral health-related quality of life. In fact, in our study population, a dental midline shift of more than 3 mm was associated with a significant decrease in oral health-related quality of life. A possible explanation for this clear association is an increased sensitivity of patients to dental asymmetries [24, 25, 38]. Several studies have already derived thresholds for the perception of midline shift. For example, laypersons recognized a midline shift of 2 mm or more [8, 35, 40]. Another study reported that a dental midline deviation of 2.92 ± 1.1 mm was no longer considered acceptable [38]. However, none of these studies investigated the extent to which these asymmetries influence oral health-related quality of life. Our results suggest that midline shift should play an important role in the indication for orthodontic treatment. Indeed, it should be reconsidered that international and national indication criteria should include midline shift in analogy to other parameters such as overjet. The impairment of oral health-related quality of life caused by a midline shift > 3 mm is indeed comparable to the impairment of oral health-related quality of life caused by an overjet > 6 mm. Our results therefore suggest that midline deviations > 3 mm may serve as a cut-off to define the need for orthodontic treatment of dental asymmetries.

Another finding of our study was that patients’ perceptions of extraoral asymmetries did not correlate with objective measurements of facial morphology using 3D scans. This is an important finding, as numerous studies have shown that facial asymmetries are very common in the general population [13, 28, 37]. However, gender seems to be a determining factor in the perception of these asymmetries. Indeed, in our study, girls were more critical in the assessment of their asymmetry. However, in line with previous studies, we found no gender differences in the objectively measured facial symmetry [13, 28]. Thus, self-perception of extraoral asymmetries is a multifactorial and multidimensional process that cannot easily be objectified by morphological measurements. In addition to psychological and cognitive factors, phenotypic and metric features, such as distortion by mirrors and photography, are also important [17, 21]. Jáuregui-Lobera et al. also demonstrated a generally higher level of dissatisfaction in body perception among female adolescents [16], while Kwak et al. found that women and men use different cues/criteria to assess/perceive asymmetry [19]. This complicates orthodontic (or even orthognathic) treatment of patients with self-perceived facial asymmetry, as patients may still perceive themselves as asymmetrical even after successful correction according to objective criteria.

However, this does not mean that the correction of facial asymmetries should not be an important goal of orthodontic treatment. In fact, in this study we found a significant correlation between dental midline deviations and facial asymmetries, as there was a significant correlation between the dental midline difference and a lateral gonion deviation (x-axis). Similar results have already been shown by Thiesen et al. in their study [36]. Lum et al. also showed greater asymmetries in three-dimensional space along the x‑axis than along the z‑ and y‑axes [22]. These results suggest that dental midline shifts are often functional in origin (e.g. due to a lateral forced bite of the lower jaw) and should be corrected as early as possible before they affect facial growth.

A limitation of this study in this respect was the lack of a more detailed examination of the aetiology of the dental midline shift. For example, it would have been interesting to obtain more information through intraoral scanning about other types of malocclusion (e.g., crowding, lateral crossbites) that may lead to midline shift. However, we could at least rule out that caries-related tooth loss was the main cause of the midline shift, as we did not observe any correlation between the dmft value and the dental midline displacements between the maxillary and mandibular arches (Table 1). Another limitation of this study was that self-perception of dental appearance/malocclusion is multifactorial and possible factors such as socioeconomic status and peer group were not investigated in this study [6, 23]. In fact, up to 50% of a patient’s treatment needs are thought to be unrelated to actual measurable abnormalities [20]. It is therefore all the more remarkable that in this study we were able to establish a clear correlation between dental a midline shift and psychosocial and emotional well-being in children and adolescents.

## Conclusion

Our results show that the perception of facial asymmetry was highly subjective and could not be objectified by stereophotogrammetric analysis using a facial scan. Therefore, clinicians should be cautious if the perception of extraoral asymmetries is the main reason for patients to undergo orthodontic treatment (especially in combination with orthognathic surgery).

On the other hand, the quantitative measurement of a midline deviation seems to be a valid parameter. A midline deviation of more than 3 mm had a negative impact on the psychosocial and emotional well-being of children and adolescents. For each additional millimeter of dental midline deviation, the impairment increased by 1.04 points on the quality of life scale (Table 4). As this parameter can be easily measured at the initial clinical diagnosis, it should be included in the assessment of the need for orthodontic treatment.

## Supplementary Information

Supplementary Fig. 1
