# Supplementary material for: Perception of facial and dental asymmetries and their impact on oral health-related quality of life in children and adolescents
Source: J Orofac Orthop. 2023 Aug 28;86(3):137–44. doi: 10.1007/s00056-023-00490-2 (PMC12043726; doi:10.1007/s00056-023-00490-2)
Supplement: Supplementary file 1 — Supplementary Fig. 1 [file 56_2023_490_MOESM1_ESM.pdf]

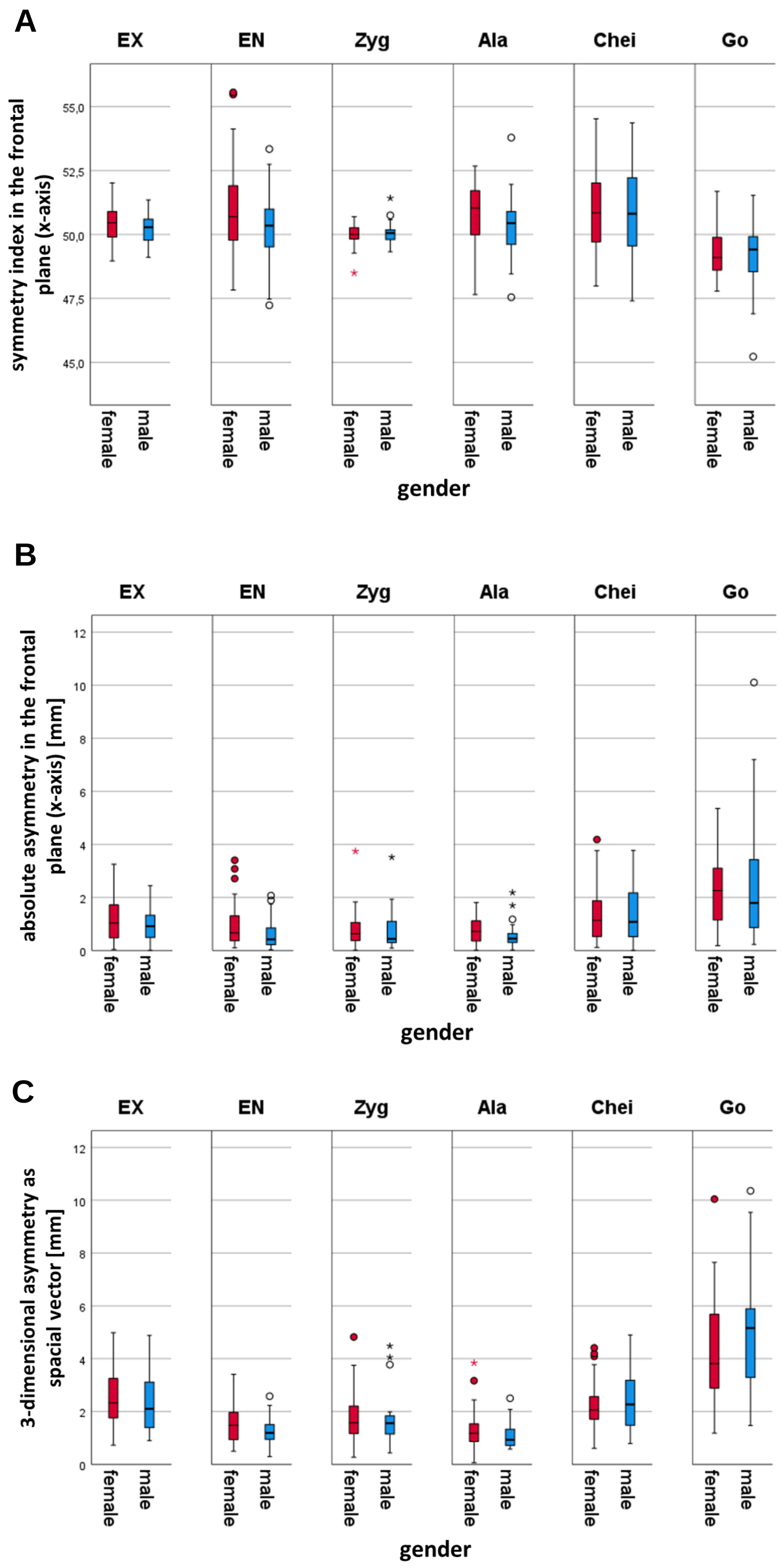

Supplementary Figure 1 Results of the 3D face scan analyses.

(A) Symmetry indices in the frontal plane (x-axis) as a function of gender. **(B)** Absolute/metric asymmetry in the frontal plane (x-axis) as a function of gender. **(C)** 3D asymmetry as a spatial vector of landmarks as a function of gender
